# Supplementary material for: Genetic and Chemical Controls of Sperm Fate and Spermatocyte Dedifferentiation via PUF-8 and MPK-1 in Caenorhabditis elegans
Source: Cells. 2023 Jan 28;12(3):434. doi: 10.3390/cells12030434 (PMC9913519; doi:10.3390/cells12030434)
Supplement: Supplementary file 1 [file cells-12-00434-s001.zip › cells-2140068-supplementary.pdf]

Supplementary Information for

Genetic and Chemical Controls of Sperm Fate and Spermatocyte Dedifferentiation via PUF-8 and MPK-1 in *Caenorhabditis elegans*

Youngyong Park[1,4], Matthew Gaddy[1,4], Moonjung Hyun[2], Mariah E. Jones[1], Hafiz M. Aslam[1], Myon Hee Lee[1,3,5]

[1]Division of Hematology/Oncology, Department of Internal Medicine, Brody School of Medicine at East Carolina University, Greenville, NC 27834, USA
[2]Biological Resources Research Group, Bioenvironmental Science & Toxicology Division, Korea Institute of Toxicology, Gyeongsangnam-do, 52834, South Korea
[3]Department of Biology, East Carolina University, NC 27858, USA
[4]These authors contributed equally to this work

Table S1. ***C. elegans*** **strains used in this study**

Strain	Genotype	Citation
N2	*wild-type (Bristol strain)*	[1]
JK3231	*puf-8(q725)*	[37]
AH102	*lip-1(zh15)*	[75]
JK3961	*puf-8(q725)/mIn[mIs14 dpy-10(e128)]; lip-1(zh15)*	[23, 25]
SD939	*mpk-1(ga111) unc-79(e1068)*	[46]
JK3997	*puf-8(q725)/mIn[mIs14 dpy-10(e128)]; lip-1(zh15); mpk-1(ga111) unc-79(e1068)*	[25]
JK816	*fem-3(q20) gain-of-function*	[38]
MHL58	*puf-8(q725)/mIn[mIs14 dpy-10(e128)]; fem-3(q20gf)*	This study
DG3913	*tn1541[GFP::tev::s::lin-41]*	[40]
MHL106	*tn1541[GFP::tev::s::lin-41]; puf-8(q725)/mIn[mIs14 dpy-10(e128)]; fem-3(q20gf)*	This study

Table S2. Antibodies used in this study

**Antibody against**	**Resource**
MSP (a marker for sperm fate)	DSHB (Cat#: 4A5)
HIM-3 (a marker for meiotic cells)	NOVUS (Cat#: 53470002)
GFP (Green Fluorescent Protein)	Abcam (Cat#: Ab290)
DP-MAPK (YT)	Sigma Aldrich (Cat#: M8159)
pERK1/2	Cell Signaling (Cat#: 9101)
ERK1/2	Cell Signaling (Cat#: 9102)
Tubulin	Sigma Aldrich (Cat#: T8203)

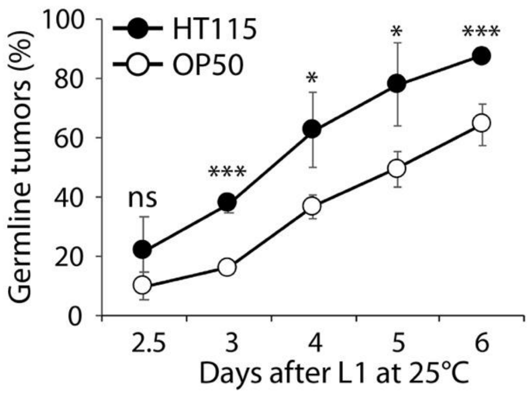

Figure S1. The effect of bacterial foods on spermatocyte dedifferentiation-mediated tumorigenesis in *puf-8(q725); fem-3(q20gf)* mutants at 25°C. The *puf-8(q725); fem-3(q20gf)* mutants fed HT115 *E. coli* had significantly more germline tumors than those fed OP50 *E. coli*. ***, p<0.001; **, p<0.01; *, p<0.05; ns, not statistically significant.
